# The Perceptions and Needs of French Parents and Pediatricians Concerning Information on Complementary Feeding

**DOI:** 10.3390/nu13072142

**Published:** 2021-06-22

**Authors:** Sofia De Rosso, Camille Schwartz, Pauline Ducrot, Sophie Nicklaus

**Affiliations:** 1 Centre des Sciences du Goût et de l’Alimentation, AgroSup Dijon, CNRS, INRAE, Université Bourgogne Franche-Comté, F-21000 Dijon, France; sofia.derosso@inrae.fr (S.D.R.); camille.schwartz@inrae.fr (C.S.); 2 Santé Publique France, French National Public Health Agency, F-94415 Saint-Maurice, France; pauline.ducrot@santepubliquefrance.fr

**Keywords:** child feeding guidelines, health communication, infant feeding, complementary feeding information, parents’ information sources, parenting, public health, pediatricians

## Abstract

Appropriate complementary feeding (CoF) is the key to preventing childhood obesity and promoting long-term health. Parents must be properly informed through the CoF process. Pediatricians have opportunities to interact with parents during the CoF transition and influence parental feeding decisions. They can convey public health nutrition messages to parents. With the release of new CoF recommendations in France in 2019, and from the perspective of their conversion into official public health communication material, the aim of this study was to explore parents’ and pediatricians’ perceptions and needs regarding information on CoF. Two online surveys were disseminated to gather information on CoF communication and guidance: one for parents (n = 1001, January 2020); one for pediatricians (n = 301, October 2019). The results showed that the importance of CoF for children’s healthy growth was well recognized by both parents and pediatricians. Parents acknowledged pediatricians as the most influential source of advice; and pediatricians were aware of their responsibility in counselling parents on CoF. However, pediatricians neglected the fact that parents gave high trust to their personal network when looking for advice. The Internet was a well-recognized source of information according to all. Diverging from what pediatricians considered useful, parents were interested in practical advice for implementing CoF. This study highlights common expectations and points of divergence between parents’ needs and pediatricians’ perceptions of those needs with regard to CoF information.

## 1. Introduction

During the first few months of life, an infant faces a phase of rapid growth, which is largely determined by early childhood and complementary feeding (CoF) practices. CoF is defined as the period when solid foods start to be introduced in an infant’s diet [1]. Inappropriate CoF can have serious implications for the healthy growth of the child and for the development of healthy eating habits [2]. Proper infant feeding gives the right footprint to a healthy process of development for the child, reducing the risks for non-communicable chronic diseases later in life [3,4]. During this period, parents are the key players in shaping the eating behaviors of their children [5], but they also have to deal with a phase of learning and upheaval at the emotional and physical level. In fact, the transition to parenthood can affect what new parents perceive as healthy eating behavior, accentuating their stress around their feeding practices [6]. High levels of parenting stress have been associated with more authoritarian parenting styles, which in the feeding context emerges in a decreased ability to interpret the child’s satiety cues, the use of more controlling child-feeding practices (e.g., pressure to eat) and offering less healthy foods to the child [7,8]. Overeating and emotional eating in children have indeed been proven to result from higher levels of parenting stress, whereas mindful parenting and mindful feeding were positively associated with healthier eating habits [9,10]. The strong concern of not knowing how to behave, and the high level of stress due to new parenting situations, can lead parents to beg for any kind of advice regarding how to care for and feed their children, thereby lowering the degree of rigor demanded of the information [11]. New parents look for information on infant feeding via many different sources—from the Internet, media, books and magazines to family, friends and healthcare professionals, among whom are pediatricians [12,13]. The feeling of reluctance that hovers around the introduction of the first solid foods and food pieces is high in some parents [14]. The confusion is raised by the fact that guidelines on infant feeding may change and evolve, in relation to the increasing evidence that centralizes the role of the first 1000 days of life of a child as a cornerstone for the early prevention of obesity [15,16].

During the last few years, in Europe and all over the world, countries have released different guidelines to properly inform parents and healthcare professionals on infant feeding practices likely to optimize short and long-term health. However, in many cases, the feeding behavior of parents is not fully in compliance with official feeding recommendations [17,18,19]. Among the many factors accounting for this gap there is, for example, the fact that national guidelines can be incomplete or not updated—some emerging evidence-based topics might be poorly covered (e.g., the introduction of different textures and how to cope with food refusal) [20]. Moreover, mothers often perceive guidelines as something divergent from what they consider the “reality” of the CoF process, and therefore they allow themselves a certain degree of flexibility in following what is recommended [21]. Considering the fact that early eating habits typically have long-lasting impacts throughout childhood and may ultimately turn into adult eating patterns [22], supporting parents and guiding them early to boost children’s healthy development is paramount.

To avoid the risk of leaving parents with inconsistent information, healthcare professionals have a responsibility to provide them with the best practice advice and guidance, based on the latest scientific evidence. In France, when the child is aged between 0 and 16, parents are encouraged to take him/her to several free and mandatory consultations with healthcare professionals, and the majority of these visits take place in the first three years of life [23]. Among all the healthcare professionals, pediatricians have one of the most important roles; they interact with parents during the CoF transition, and therefore they are in a convenient position to provide advice to support parental decisions when it comes to feeding matters. Pediatricians are trusted by parents in France, as shown by the Nutri-Bébé 2013 study: 58% of mothers of children aged between 15 days and 35 months appealed to medical advice for feeding their children [24]. In France, the official updated recommendations for feeding children aged 0 to 3 years old have recently been published [25,26] to replace the previous recommendations dated back to 2004. Simultaneously investigating the perceptions and needs of parents and pediatricians regarding CoF information could help to build an evidence-based strategy for developing a targeted health communication program.

To allow public health stakeholders to develop a communication strategy to effectively spread the latest official recommendations on infant and young children feeding, this study aims to provide an overview of the current situation, in France, in terms of perceptions towards CoF and the CoF information needs of parents and pediatricians. Therefore, we first describe parents’ and pediatricians’ perceptions and needs regarding the available information on CoF, and subsequently investigate parental information-seeking strategies and behaviors (sources used to gather CoF information, content searched for, etc.) seen from both parents’ and pediatricians’ perspectives.

## 2. Materials and Methods

### 2.1. Web Surveys and Participant Sampling

Two online surveys were prepared and administrated for each target group separately. The parent survey targeted a French representative sample of 1000 parents of children <4 years old and was prepared with the help of a private research and consulting firm (BVA). It was open for completion from the 10 to the 29 of January 2020. The sample size of 1000 was defined a priori, and it was considered representative for our purposes according to previous studies conducted by BVA on similar subpopulations. The sample of parents aimed to represent the French population of young parents. The quota method was applied to ensure the representativeness of the sample, and the general population census was used for data calibration [27]. The following variables were controlled for with quotas: age of the parent; profession of the household reference person (i.e., the parent with the highest salary); region; living area; first-time or multiparous parent. Multiparous parents (included in the study because at least one of their children was <4 years old) were asked to refer to their youngest children when answering to the questionnaire. A total of 1001 parents responded to the survey and their answers were considered for analysis. The questionnaire was divided into five sections: characteristics of the youngest child, the parents and the household; parents’ perceptions on CoF; parents’ perceptions on available CoF information; sources of information used by parents (with self-perceived influence on their feeding practices) and type of CoF information sought by parents.

The second survey was intended for members of the French Association of Ambulatory Pediatrics (AFPA, n = 1402). The AFPA is a nationwide recognized association that brings together many pediatricians (mostly in private practice) from all over France (https://afpa.org/; accessed date: 4 May 2021). The pediatricians’ questionnaire was prepared with Sphinx and remained open for completion from the 10 of October to the 7 of November 2019. Considering the total size of the population being studied (n = 1402), and according to a power calculation with a confidence interval of 95% and a margin of error of 5%, the required number of respondents to reach was equal to 302 [28]. Answers were obtained from 318 healthcare professionals, the 301 answers from pediatricians were considered for the analysis (the few general practitioners who answered were excluded in favor of a more homogeneous sample). The questionnaire consisted of four parts: demographical data, attitudes and perceptions regarding CoF and CoF information, current practices regarding communication with parents about CoF and new plans/suggestions for implementing CoF communication with parents. For both questionnaires, all the answers were mandatory and the inclusion of participants ended when the sample sizes had been reached.

The original versions of the questionnaires (in French), with translations to English, are presented as Supplementary Materials S1 and S2, for parents and pediatricians, respectively. The questions and the lists of answers (multiple choice) were developed based on previous studies [12,29], while considering the advice of public health experts and BVA for the parents’ survey. The pediatricians’ questionnaire was piloted with a small network of healthcare professionals (from the Perinatal Network of Burgundy, n = 200; n = 33 of participants answered the pilot survey). The parents’ questionnaire was pretested by BVA with their panelists. Both questionnaires included closed-ended multiple choice questions, with one or check-all-that-apply answer options; Likert scale and demographic questions; and one optional open question for the pediatricians’ questionnaire asking for additional suggestions to implement the content or format of the material to facilitate communication on CoF with parents. The Likert scale used was differently in the two surveys. A 5-point scale (strongly agree; agree; neither disagree nor agree; disagree; strongly disagree) was used to ask pediatricians if they agreed with statements regarding their attitudes and perceptions on CoF in general, and with the available CoF information they give to parents. After consultation with BVA, it was decided to remove the “neutral” option from the parents’ survey; this decision was made in order to gather specific and stronger opinions from parents, but ran the risk of forcing respondents with no opinion to make decisions. Therefore, a 4-point scale (strongly agree; tend to agree; tend to disagree; strongly disagree) was used to ask parents if they agreed with some statements regarding perceptions around CoF and available CoF information. Statements requiring an answer on a Likert scale were considered as discrete variables and were grouped in “agree” and “disagree” (and “neutral” for the 5-point Likert scale of the pediatricians’ survey) for the description of the results.

The parents’ and pediatricians’ surveys addressed many identical issues, with questions formulated almost in the same way, but there were also some differences. In the parents’ questionnaire, parents were asked to report how much their sources used to gather CoF information influenced them (continuous scale from 1 to 10, with 1 meaning the source was not at all influential and 10 meaning it was strongly influential) in making decisions on how to feed their youngest children. For the majority of the questions in the parents’ questionnaire, we referred to “feeding children aged 0–3 (other than milk feeding), including complementary feeding” and specified the definition of CoF in order to avoid misunderstandings. In the pediatricians’ questionnaire, specific questions addressed, for example, how they obtained information on CoF in the last two years and who they thought formulated official infant and young children feeding recommendations in France.

### 2.2. Ethical Consideration

The studies were conducted according to the guidelines laid down in the Declaration of Helsinki. This kind of research in France does not require mandatory approval from an ethics committee. For the parents’ study, a panel of eligible respondents was surveyed and ethical measures were undertaken according to BVA’s procedures. Written informed consent was obtained from all subjects and the panel of participants involved in the study was declared to the appropriate committee (CNIL—Commission nationale de l’informatique et des libertés) for data protection. The processing of the personal data of BVA panel members was carried out in accordance with the European regulation “General Data Protection Regulation (GDPR)”. All participants were assigned unique identifiers by BVA. The unique identifier allowed registering in the panel and it is essential for BVA to manage the contacts with panelists. Researchers did not have access to identifying panelist data. Small incentives were given to parents for participation. For the pediatricians’ study, researchers never had access to any personal data of the respondents, who accepted to participate voluntarily and anonymously.

### 2.3. Data Analysis

For all statistical analyses, R version 3.6.1 was used [30]. Frequencies, percentages and means ± SDs were used to describe the results. The quota sampling method ensured the representativeness of the parents’ sample for the general population. For the parents’ sample, a Friedman test was carried out to see if the sources of information had different perceived levels of influence. Chi-square tests were calculated to check whether the pediatricians who answered the survey were a representative sample of the AFPA pediatricians regarding age and gender. An independent sample *t*-test was used to compare means, and the test for homogeneity was used to draw a conclusion about whether the distribution of responses was the same in the two populations. The level of significance was set at *p* < 0.05.

## 3. Results

The main findings reported in this paper provide an overview of perceptions and needs regarding information on CoF, accounting for the points of view of French parents and pediatricians. For example, regarding perceptions: Is CoF important for the healthy development of the child? Are parents satisfied with the available information? Do pediatricians think they have the means to start discussing CoF with parents during consultations. Regarding needs: When is the information needed? what type of content do parents look for? What do pediatricians think is important to tell to parents? Which sources are used and influential to give information on CoF?

### 3.1. Study Populations

The study populations are described in Table 1. Answers were obtained from a total of 1001 parents, representative of the French parents’ population with at least one child <4 years, according to the quota sampling method. Information was available regarding age and gender of the pediatrician members of AFPA. The sample of pediatricians (n = 301) was representative for age, with 47% of members being less than 50 years old, 27% 51 to 60 years old and 26% over 60 years old. Regarding the gender, there was an overrepresentation of female pediatricians in our sample (80%) compared to the general AFPA population (75%).

### 3.2. Perceptions of Parents and Pediatricians towards CoF and Feeding Children Aged 0–3

All parents were aware of the importance of CoF for their children’s current and future health and growth (99%); 77% even thought it was very important. In addition, all parents (99%) knew that this period is important for developing health-favoring eating habits in children, and 79% thought it is very important. Overall, the period of CoF was positively experienced by parents. Thus, 91% considered that it went well. Nevertheless, for 35% of parents, CoF was or had been a cause of concern.

The importance of CoF for the development of young children was well recognized by all pediatricians (99%), as was the need to advise parents on the subject in order to help them adopt healthy feeding practices for their children (93%; 7% were neutral); 68% thought this was very important. Ninety-nine percent of pediatricians were confident in their role of advising parents about CoF, and 82% of them thought they had enough knowledge about CoF and infant feeding. All pediatricians believed that advising parents on CoF was one of their responsibilities; 93% of them always gave advice on this subject during medical consultations, compared to 7% who gave advice only when they recognized it was necessary.

### 3.3. When Did Parents Look for Information on CoF and When Did Pediatricians Provide Advice?

On average, parents began to learn about CoF when children were five months of age (4.7 ± 3.7). When the child was less than six months old, during medical follow-up consultations with the child, 52% of pediatricians spontaneously broached the subject of CoF and 54% of parents asked questions or sought advice. Seventy-nine percent of interviewed parents reported they already looked for information on CoF for at least one of their children, and 73% for their last child.

Eighty two percent of pediatricians said they started giving CoF advice to parents when children were around four months of age (3.8 ± 0.8), tackling the issue of the different stages of CoF when children were 4 or 5 months of age (4.3 ± 0.7).

### 3.4. Parents’ Perceptions about CoF Information and Pediatricians’ Perception about Parental Information Needs

Eighty seven percent of parents felt they were well informed about CoF, and 87% of them were satisfied with the available information. The proportion of parents who considered themselves to be very well informed was rather small (37%); the same went for those who felt very satisfied with the information (29%) or considered the search for information to be very easy (28%). The information on the subject of CoF appeared clear to 90% of parents; that relevant to their questions was present for 87%; and it easy to put into practice for 86% of parents. However, a third of parents (34%) considered that the advice available was not always consistent and could even be contradictory. According to 32% of parents, the available advice on infant feeding could make them experience a feeling of guilt of not behaving as recommended.

Forty-one percent of pediatricians thought that parents did not want more information on CoF than what they were able to provide during consultations, whereas 31% of pediatricians thought parents wanted more information (28% were neutral regarding this topic). Although only 16% of pediatricians said they were not satisfied with the available CoF material, 55% of them said they prepared the documents on CoF to give to parents themselves. Thirty-five percent of pediatricians thought that the different economic and cultural situations of families were not sufficiently taken into account in the information and communication materials available to parents (35% were neutral; 30% thought differences were considered).

### 3.5. Type of Content Parents Are Looking for Versus What Pediatricians Thought Is Important for Parents to Know

**Figure 1:**
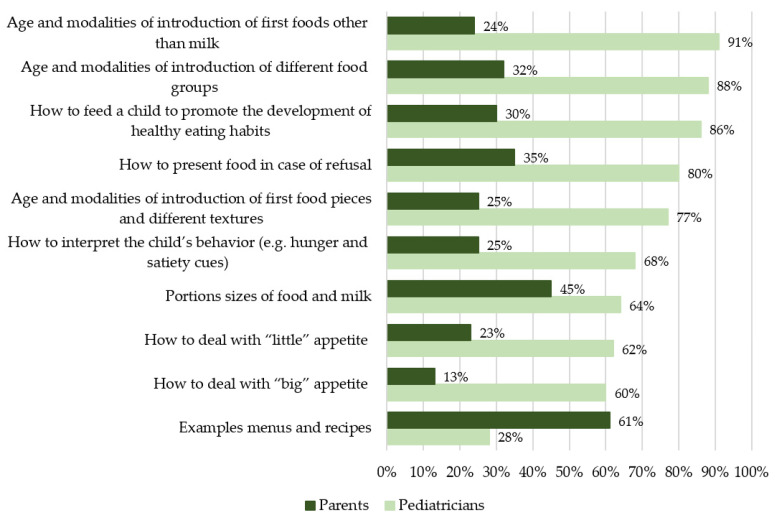
Levels of importance of various topics related to CoF according to French parents (n = 1001) and pediatricians (n = 301). Ranked by decreasing importance for pediatricians.

Figure 1 shows that parents found particularly useful information on very concrete topics, such as examples of recipes or menus (61%), quantities and sizes of food and milk portions (45%), how to present food in case of refusal (35%), ages of introduction for different food groups (32%) and strategies to feed the child to promote the development of healthy eating habits (30%).

Concerning the information to be transmitted to parents about CoF, pediatricians thought that the most important topics were: the age of start to CoF (age and modalities of introduction of first foods other than milk—91%), the ages at which the different food groups must be introduced (88%), how to feed a child to promote the development of healthy eating habits (86%) and presentation strategies in case of food refusal (80%). Only 28% of pediatricians thought it was important to provide more information on recipes and menus, a topic widely sought for by parents (Figure 1). For each question/topic, the percentages were significantly different for parents and pediatricians (*p* < 0.001).

### 3.6. Sources Used by Parents to Learn About CoF (with Their Degrees of Influence), and Sources Pediatricians Thought Were Relevant for Parents

**Figure 2:**
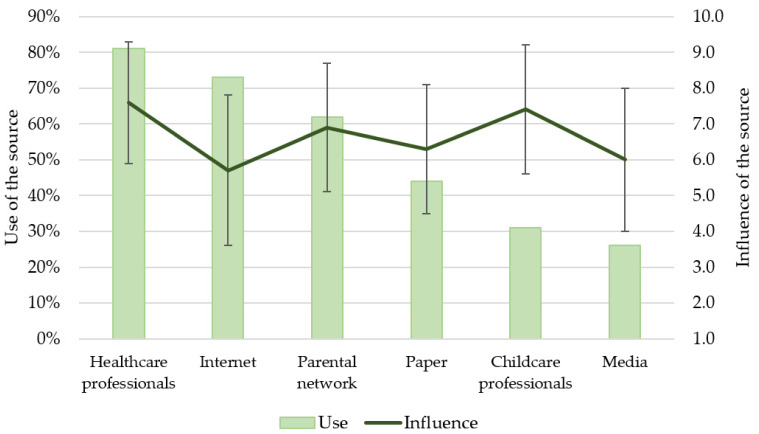
Parental perception of the influence of each information source on their feeding practices, and degree of usage for each source. Left axis: percent of use among all respondents (n = 1001); right axis: self-perceived influence (Mean +/−SD; scale from 1 to 10) of the information source among those who reported to use that corresponding source.

Figure 2 shows the use of the different sources of information in relation to their influences on parents in terms of decision making when feeding their children. Healthcare professionals were a source of information for 81% of parents, and their degree of influence was the highest (7.6 ± 1.7/10). Among healthcare professionals, the pediatricians and general practitioners were positioned as the main vectors of information (49% and 46%). The Internet in the broad sense (including websites, blogs, social networks and applications) turned out to be the second most used source of information; 73% of parents resorted to these online means. However, in spite of the widespread use, the mean level of influence for the Internet was the lowest (5.7 ± 2.1/10). The parental network was globally identified as a source of information by 62% of parents, with a mean influence of 6.9 ± 1.8/10. Paper documents were a source of information for 44% of parents, and of relatively low influence (6.3 ± 1.8/10). Early childhood professionals were among the sources of information that parents used the least (31%), but they had a strong influence on parents (7.4 ± 1.8/10). Only 26% of parents referred to the media (television, radio) and the mean influence of this source of information was 6.0 ± 2.0/10. There was a statistically significant difference in self-perceived influence depending on the information source used (Friedman test: χ2(5) = 1191, *p* < 0.001).

For pediatricians, the most effective tools to grab the attention of parents were websites (for 73% of respondents), paper brochures (59%) and smartphone or tablet applications (57%). Only 13% of pediatricians thought that parents relied on their personal networks to gather advice, and therefore they underestimated the usage of this source. According to 98% of pediatricians, the bodies responsible for formulating official recommendations concerning CoF in France were the pediatrician professional organizations; only 56% of them recognized Public Health France, the French public health agency, as an official source in charge to inform the general public about feeding recommendations.

### 3.7. Appropriate Communication Formats According to Parents

As shown in Table 2, for 79% of parents, the most appropriate communication format was digital (applications or websites), but 58% of parents liked the paper format. Smartphone applications (43%), multi-page booklets (41%) and websites (38%) were the most suitable formats for providing information on CoF. A one-page brochure seemed less appropriate (23%).

## 4. Discussion

Two surveys were conducted in France at the end of 2019/beginning of 2020. The aim was to explore the needs and perceptions of parents and pediatricians in regard to information on CoF, in order to inform the deployment of a public health strategy to disseminate the new CoF recommendations issued in 2019. The results showed that there was full awareness of the fact that the CoF period has a strong impact on the healthy development and growth of children, and on the evolution of their eating habits, from both parents’ and pediatricians’ points of view. Nevertheless, for some parents, CoF was perceived as a cause of concern. There was clear evidence that providing CoF information to parents is essential in order to help them understand how to adopt healthy eating practices for their children, and pediatricians were well-recognized by parents as information providers. We observed that information was requested by parents and advice was given by pediatricians before the implementation of CoF at slightly different times. Notwithstanding, according to parents, only half of the pediatricians spontaneously broached the subject of CoF during medical appointments, and this is in contrast with the majority of pediatricians saying that they always gave advice on this subject during consultations. Despite the preponderant feeling of parents that they were well informed and satisfied with the information they are able to find, over one third of them perceived the available advice as contradictory and inconsistent.

The parental perception of receiving conflicting information highlights the confusion that parents can experience when they are put in the position of deciding which advice to follow regarding their child feeding. Mothers can be pushed to the point of not relying to any feeding guidance, but just on their own instinct and on the fact that “all babies are different,” and they may consequently adapt their feeding practices to what they think is the best for their babies [31]. Moreover, our analysis showed that, according to more than one third of the interviewed pediatricians, the information and communication material available for parents may be culturally inappropriate, with approaches that may not account for different socio-economical situations of families. The unsuitability of existing CoF communication means for the needs of disadvantaged populations frequently emerged in literature. Parents complain, for example, that healthcare professionals do not know their culture and traditions, and therefore they trust family members better [32]. Additionally, mothers who, for some reason (e.g., medical concerns: crying, spitting up and waking), are unable to follow the infant feeding recommendations are not inclined to seek advice from healthcare professionals even in case of difficulty, as they do not feel understood by them [33].

Even though these previous studies suggested a conflicting and unstable level of trust in healthcare professionals’ guidance, our results showed that pediatricians and general practitioners are the most used source of information for parents and the advice coming from them is the most influential in terms of feeding practices. Findings from other studies suggested that healthcare professionals’ advice was valued more if perceived as coming from personal experience or scientifically verified, up-to-date sources [34,35]. Consistently with earlier findings, parents in this survey also gave high value to the advice from family and friends, and one reason can be that they did not perceive the health professionals’ advice as practical or relevant to the needs of their children [36,37]. The social pressure from peers (family members, friends or other parents of young children in the own network), but also the existence of social norms and their influences on infants and feeding decisions are topics widely discussed in literature and might undermine adherence to recommendations [38]. Our results bring new insights about the fact that pediatricians in this study largely underestimated parents’ reliance on their personal network. This is unfortunate since, if they do not realize who parents are listening to, they cannot attempt to counterbalance the influence of these sources. Healthcare professionals should seek every opportunity to promote infant feeding recommendations during medical appointments. In doing this, it is important to try to positively involve parents, reassure them in their new roles and build confidence in a way to override social expectations or strict cultural advice.

Another aspect that might be considered is whether the advice coming from physicians is in line with infant feeding guidelines. Surprisingly 55% of pediatricians produced their own CoF materials, which can leave room to personal interpretation of public health messages. A study conducted in the South of France showed that pediatricians’ advice on CoF was only partially in line with current recommendations [39]. Moreover, frequently updated recommendations were well followed by pediatricians, but those that had not been recently updated were less well followed [39]. Contrariwise, the findings from Chouraqui and colleagues showed that French pediatricians and general practitioners give CoF advice mostly in line with the official guidelines. Despite the congruence of given advice with recommendations, parents still experience confusion due to the fact that their perceptions rarely align with what is said by doctors; that is one reason why they are still far to reaching the point where their feeding behaviors perfectly match guidelines [40].

Our results revealed that only a small percentage of pediatricians were not satisfied with the available material on CoF, but over half of them declared that they prepared themselves the documents with CoF information to give to parents. Moreover, the vast majority of the pediatricians of our study could not correctly identify the body in charge of the release of official feeding guidelines, and over half of them did not know who handled the production of the related official material. The fact of preparing their own materials to distribute to their patients can be seen as a sign of mistrust in institutions or a lack of awareness of the roles of various public health stakeholders. Knowing who is developing the material could increase pediatricians’ level of trust and encourage them to use official material instead of creating and distributing their own. It is important to create a nationally unified strategy to communicate in two directions: directly with the general population, via accessible information disseminated by appropriate means, but also with healthcare providers, such as pediatricians who are in direct contact with the parents and work on the front line to spread health-related information.

During the last few years, research has incrementally focused on the different aspects of parenting, including feeding [41,42,43]. It is well acknowledged that becoming a parent, especially for the first time, is a life changing experience that brings together joy and challenges, along with the stress of not fitting into this new role [44,45]. The reality of the early parenting journey is complex, and it is difficult to generalize as there is not a unique way to face it [46]. The fact of dealing for the first time with a new situation, for example, CoF, brings parents to search for advice. Parents might feel overwhelmed with all the information they can easily access via many sources and with the influences and pressure coming from their social environments, and this can be one other factor that contributes to increasing their level of confusion [31,47]. The parents in our study acknowledged mainly referring to healthcare professionals for trustworthy advice. The Internet was also a recurrent source of information, even if its influence was low. Being able to find easily trustworthy health related advice can help parents to make use of the powerful tools of information and communication to better cope with the challenges they face every day. In this light, exploiting the resources made available by new technologies is essential, as we saw in our study how much parents made use of the Internet and applications, for example.

Public health stakeholders in charge should prepare the communication strategy for the new recommendations in accordance with the different needs of parents and pediatricians in terms of CoF information. Pediatricians, but also other healthcare professional figures that have the opportunity to be in contact with new parents during the early-child feeding period, should be considered as central for the dissemination of the new recommendations, and training courses in this regard could be organized at the national level. Since pediatricians are the first relay of advice for parents, it is important that they can align with parents’ demands of information. Parents look for practical information, such as menus and recipes to propose to the children when introducing new foods into their diets, whereas pediatricians think the most important information to give to parents is the ages of introduction for the different foods and food groups. A double-pronged strategy could be used, allowing, for example, material with detailed information for pediatricians and material with more practical tips for parents. Translations in other languages, or offering some specific examples of menus and recipes adapted to different “cuisines” could also account for cultural differences. Moreover, to reach optimal diffusion of official information, it is paramount that pediatricians recognize the bodies in charge of formulating and spreading official recommendations. Pediatricians’ professional organizations could be relevant in this direction in order to focus the attention and augment the level of trust of the professionals towards the right stakeholders in charge to provide evidence-based information to the different members of the public. Official public health agencies and institutional logos could be associated with the information given by pediatricians’ professional organizations in a way to improve visual familiarization when talking about official recommendations via different channels.

Some strengths and limitations must be considered alongside our study. First of all, both surveys allowed us to collect quantitative data, but we could not explore further motivations behind the answers of the participants. Moreover, we aimed at collecting similar information within two different populations (parents and pediatricians), but we had to adapt some questions, which therefore might make slightly difficult the direct comparison of some topics. One strength of this study lies in the utilization of large samples; the parents’ sample in particular is nationally representative of the French population of young parents, and it accounts for the point of view of primiparous and multiparous parents, something not common in the literature. Since, to the best of our knowledge, there were no existing studies focusing exclusively on CoF information needs in France, this double survey provides important data by seeking the views of both parents and pediatricians, thereby providing opportunities to build on these findings from the perspective of designing a national communication strategy in an appropriate, timely manner.

When implementing public health recommendations regarding infant feeding, attention should be paid to relays of information, such as pediatricians, who are conscious of their role in this regard, and whom parents trust. In this process, the present data suggest that it is important to ensure that parents have access to clear and non-contradictory messages, in line with updated recommendations.

## 5. Conclusions

Our results highlighted some common points and divergences in perceptions and needs of French parents and pediatricians regarding CoF information. In particular, parents have the perception that they are provided with inconsistent and contradictory CoF information; they need clear guidance from the sources they use and that influence them the most (e.g., healthcare professionals). Moreover, advice should be provided to parents in a non-pressuring but engaging manner, in order to avoid the establishment of feelings of inadequacy or guilt. Relevant and easy to understand messages, given with consistency, might make CoF information less confusing for parents, thereby providing more realistic and acceptable advice, which might increase compliance with the official recommendations.

Pediatricians acknowledge the need for CoF informational material able to provide support accounting for family and cultural differences. Pediatricians should be aware of the influence of parents’ personal networks and the need for more practical tips. It is essential that pediatricians understand the parental information demands in order to deliver timely and appropriate CoF messages based on updated recommendations.

Effort is demanded from the public health sector in order to build a strategy to spread the new infant and child feeding recommendations via the means of informational material adapted to meet parents’ and pediatricians’ needs. In particular, pediatricians’ needs and perceptions must be considered, given the importance that parents confer to them. Moreover, given the coexistence of different sources of information that can influence parents, it is also important that the advice given by pediatricians is consistent with the official recommendations and that pediatricians recognize the official bodies in charge of the communication material. This strategy should focus on the provision of CoF information via simplified and coherent messages in order to facilitate the communication between parents and pediatricians and reduce parental confusion around feeding recommendations.

## Figures and Tables

**Table 1:** Characteristics of French parents and pediatricians who responded to the survey.

Parents (N = 1001)			Pediatricians (N = 301)		
Characteristics		N (%)	Characteristics		N (%)
Age	Less than 35 years old	604 (60)	Age	≤30 years old	12 (4)
				31–40 years old	78 (26)
				41–50 years old	45 (15)
				51–60 years old	75 (25)
	35 years old and more	397 (40)		≥61 years old	91 (30)
Age of the youngest child	<12 months	277 (28)	Years of working experience	0–10 years	80 (26)
	12–23 months	260 (26)		11–20 years	57 (19)
	24–35 months	260 (26)		21–30 years	75 (25)
	≥36 months	204 (20)		More than 30 years	89 (30)
Gender	Men	198 (20)	Gender	Men	60 (20)
	Women	803 (80)		Women	241 (80)
Parity	Primiparous	388 (39)	Having children	Yes	260 (86)
	Multiparous	613 (61)		No	40 (14)
Education level ^1^	<A level	179 (18)	Working area	Rural	29 (10)
	≥A level	822 (82)		Urban	272 (90)
Socio-professional category of the household reference parent ^2^	High	456 (46)	/	/	/
	Low	501 (50)			
	No occupation/retired	44 (4)			
Self-perception of household financial situation ^3^	Good	457 (46)	/	/	/
	Difficult	535 (53)			
	No answer	9 (1)			

^1^ A level corresponds to the diploma obtained after completion of upper secondary school (equivalent to 12 years of formal education in France). ^2^ I.e., the parent with the highest income. The socio-professional category was high (liberal profession, entrepreneur, executive or higher intellectual profession), intermediate or low (laborers and clerks) or no occupation/retired (including also students). ^3^ Parents were classified as having a good financial situation when they perceived they were comfortable or okay with it. The other parents were classified as being in a difficult financial situation when they had a perceived uncomfortable situation imposing to pay attention to their budget, or making it difficult to reach the end of the monthor forcing them to take out debts (Supplemental Material S1, question 28, modalities 3, 4, 5).

**Table 2:** The appropriate means to receive CoF information according to parents (N = 1001).

	Parents (N = 1001)
Communication Format	N (%)
**Subtotal Website or application**	79 (790)
Smartphone application	43 (434)
Websites	38 (383)
Interactive space on the Internet (Blogs, chats, forums…)	35 (347)
**Subtotal paper format**	58 (584)
Multi-page paper booklet	42 (417)
1-page paper brochure	23 (232)

## Data Availability

The data presented in this study are available on request from the corresponding author.

## Supplementary Materials

The following are available online at https://www.mdpi.com/article/10.3390/nu13072142/s1, Supplement Material S1: Parents’ original questionnaire with English translation, Supplemental Material S2: Pediatricians’ original questionnaire with English translation.
