# Supplementary material for: The Perceptions and Needs of French Parents and Pediatricians Concerning Information on Complementary Feeding
Source: Nutrients. 2021 Jun 22;13(7):2142. doi: 10.3390/nu13072142 (PMC8308433; doi:10.3390/nu13072142)
Supplement: Supplementary file 1 [file nutrients-13-02142-s001.zip › Supplement Material 1_parents survey.pdf]

Supplement Material 1. Parents' original questionnaire with English translation in blue.

Enquête sur les parents de jeunes enfants et la diversification alimentaire

Questionnaire

Survey on parents of young children and complementary feeding

Questionnaire

Renseignements signalétiques/quotas

Identifying information/quotas

Z9. Combien d'enfants avez-vous ?

Z9. How many children do you have?

Si vous n'en avez pas, indiquer 0

If you do not have any child write 0

/__/__/ enfant(s)

/__/__/ children

Z10. Précisément quel est le mois et l'année de naissance ainsi que le sexe de chacun de vos enfants ?

Z10. Precisely which is the month and year of birth as well as the sex of each of your children?

a) 1er enfant : Mois : /__/__/ Année /__/__/__/__/ sexe : 1. Homme 2. Femme

b) 2ème enfant : Mois : /__/__/ Année /__/__/__/__/ sexe : 1. Homme 2. Femme

c) 3ème enfant : Mois : /__/__/ Année /__/__/__/__/ sexe : 1. Homme 2. Femme

d) …

a) 1st child: Month: / __ / __ / Year / __ / __ / __ / __ / sex: 1. Male 2. Female

b) 2nd child: Month: / __ / __ / Year / __ / __ / __ / __ / sex: 1. Male 2. Female

c) 3rd child: Month: / __ / __ / Year / __ / __ / __ / __ / sex: 1. Male 2. Female

d)…

Z1. Êtes-vous :

Z1. Are you :

1. Un homme
2. Une femme

1. A man
2. A woman

Z2. Quel est votre âge ?

Z2. How old are you?

/__/__/ ans

/__/__/ years old

Z0a. Merci d'indiquer le code postal de votre commune

Z0a. Please indicate the zip code of your municipality

/__/__/__/__/__/

Z0b. Dans quelle commune habitez-vous ?

Z0b. In which municipality do you live?

Z3. En ce moment, quelle est votre activité principale ?

Z3. Cuurently what is your main activity?

1. Salarié du secteur privé
2. Salarié d'une entreprise publique ou nationale
3. Salarié du secteur public
4. A votre compte
5. A la recherche d'un premier emploi
6. A la recherche d'un emploi (vous avez déjà travaillé)
7. A la retraite
8. Au foyer
9. Elève ou étudiant
10. Dans une autre situation

1. Private sector employee
2. Employee of a public or national company
3. Public sector employee
4. On your own account
5. Looking for a first job
6. Looking for a job (you have already worked)
7. Retired
8. stay at home mother/father
9. Student
10. In another situation

Z3a. Actuellement, travaillez-vous à…

Z3a. Currently, do you work…

1. Temps complet
2. Temps partiel

1. Full-time
2. Part-time

Z3b. Quelle est votre profession, votre catégorie socio-professionnelle ?

Z3b. What is your profession, your socio-professional category?

Z3b. Avant d'être au chômage, quelle était votre dernière profession, votre dernière catégorie socio-professionnelle ?

Z3b. Before being unemployed, what was your last occupation, your last socio-professional category?

1. Agriculteur exploitant
2. Artisan petit commerçant
3. Chef d'entreprise de plus de 10 salariés
4. Profession libérale (SAUF paramédical)
5. Professeur / profession scientifique
6. Cadre et autre profession intellectuelle supérieure
7. Contremaître, agent de maîtrise, profession paramédicale, technicien
8. Instituteur
9. Employé
10. Personnel de service
11. Ouvrier / Ouvrier agricole
12. Retraité
13. Élève / étudiant
14. Autre inactif

1. Farmer-operator
2. Artisan small trader
3. Company manager with more than 10 employees
4. Liberal profession (EXCEPT paramedical)
5. Professor / scientific profession
6. Executive and other higher intellectual profession
7. Foreman, supervisor, paramedical profession, technician
8. Teacher
9. Employee
10. Service staff
11. Worker / Agricultural worker
12. Retired
13. Pupil / student
14. Other inactive

Z4. Êtes-vous la personne de référence du ménage ?

La personne de référence est la personne en activité au sein de votre foyer qui apporte le plus de revenus

Z4. Are you the household reference person?

The reference person is the person working in your household who brings the highest income

1. Oui
2. Non
1. Yes
2. No

Z8. En ce moment, quelle est l'activité principale de la personne de référence du ménage ?
La personne de référence est la personne en activité au sein de votre foyer qui apporte le plus de revenus
Z8. Currently, what is the main activity of the household reference person?
The reference person is the person working in your household who brings the highest income

1. Salarié du secteur privé
2. Salarié d'une entreprise publique ou nationale
3. Salarié du secteur public
4. A son compte
5. A la recherche d'un premier emploi
6. A la recherche d'un emploi (a déjà travaillé)
7. A la retraite
8. Au foyer
9. Élève ou étudiant
10. Dans une autre situation

1. Private sector employee
2. Employee of a public or national company
3. Public sector employee
4. Self-employed
5. Looking for a first job
6. Looking for a job (has already worked)
7. Retired
8. At home
9. Student
10. In another situation

Z8b. Quelle est la profession, la catégorie socio-professionnelle de la personne de référence du ménage ?
Z8b. What is the profession, the socio-professional category of the household reference person?

Z8b. Avant d’être au chômage, quelle était la dernière profession, la dernière catégorie socio-professionnelle de la personne de référence du ménage ?
La personne de référence est la personne en activité au sein de votre foyer qui apporte le plus de revenus
Z8b. Before being unemployed, what was the last occupation, the last socio-professional category of the household reference person?
The reference person is the person working in your household who brings the highest income

1. Agriculteur exploitant
2. Artisan petit commerçant
3. Chef d'entreprise de plus de 10 salariés
4. Profession libérale (SAUF paramédical)
5. Professeur / profession scientifique
6. Cadre et autre profession intellectuelle supérieure
7. Contremaître, agent de maîtrise, profession paramédicale, technicien
8. Instituteur
9. Employé
10. Personnel de service
11. Ouvrier / Ouvrier agricole
12. Retraité
13. Élève / étudiant
14. Autre inactif

1. Farmer-operator
2. Artisan small trader
3. Company manager with more than 10 employees
4. Liberal profession (EXCEPT paramedical)
5. Professor / scientific profession
6. Executive and other higher intellectual profession
7. Foreman, supervisor, paramedical profession, technician
8. Teacher
9. Employee
10. Service staff
11. Worker / Agricultural worker
12. Retired
13. Pupil / student
14. Other inactive

Z6. De combien de personnes se compose votre foyer, vous y compris ?
Z6. How many people live in your household, including yourself?
1. 1 personne

2. 2 personnes
3. 3 personnes
4. 4 personnes
5. 5 personnes
6. 6 personnes
7. 7 personnes
8. 8 personnes
9. 9 personnes ou plus

1. 1 person
2. 2 people
3. 3 people
4. 4 people
5. 5 people
6. 6 people
7. 7 people
8. 8 people
9. 9 people or more

Vécu et comportements avec l'enfant le plus jeune
Feeding behavior related to the youngest child

Cette enquête concerne principalement VOTRE ENFANT LE PLUS JEUNE. Merci de bien lire attentivement toutes les questions et d'en tenir compte lorsque vous répondez.
This survey is primarily about YOUR YOUNGEST CHILD. Please carefully read all questions and take them into account when answering.

Q1. Votre plus jeune enfant est né…
Q1. Your youngest child was born ...

1. À terme (accouchement à 37 semaines ou plus)
2. Prématuré (accouchement à 36 semaines ou moins)

1. At term (delivery at 37 weeks or more)
2. Premature (delivery at 36 weeks or less)

Q2. Votre plus jeune enfant a-t-il été allaité au sein (tire-lait inclus) ?
Q2. Was your youngest child breastfed (including fed from pumped breastmilk)?

1. Oui
2. Non

1. Yes
2. No

Q3. Combien de temps a-t-il été allaité au sein (tire-lait inclus) ?

Q3. How long has he been breastfed (including fed from pumped breastmilk)?

1. /__/ jours
2. /__/ semaines
3. /__/ mois
4. Je ne sais pas

1. /__/ days
2. /__/ weeks
3. /__/ months
4. I do not know

Q4. Est-ce que votre plus jeune enfant a commencé à manger des aliments autres que le lait ?

Q4. Has your youngest child started to eat foods other than milk?

1. Oui
2. Non

1. Yes
2. No

Q5. Actuellement, à quelle fréquence donnez-vous à votre plus jeune enfant…

Q5. Currently, how often do you give to your youngest child ...

a) des plats ou petits pots pour bébé achetés dans le commerce ?
b) des plats ou des petits pots maison que vous avez cuisinés ?

1. Systématiquement
2. Souvent
3. De temps en temps
4. Jamais

a) commercial baby food?
b) any homemade dishes or small pots that you have cooked?

1. Systematically
2. Often
3. From time to time
4. Never

Q6. Depuis la naissance de votre plus jeune enfant, lors de rendez-vous médicaux habituels avec des professionnels de santé (pédiatre, médecin, infirmier…), vous ont-ils <u>donné spontanément</u> des conseils sur l'alimentation de votre enfant (aliments autre que le lait) dont la diversification alimentaire ?

La diversification alimentaire est la période pendant laquelle des aliments autres que le lait sont progressivement introduits dans l'alimentation d'un enfant

Q6. Since the birth of your youngest child, during usual medical appointments with health care professionals (pediatrician, doctor, nurse, etc.), did they spontaneously give you advice on feeding your child (foods other than milk) including complementary feeding?

Complementary feeding is the period during which foods other than milk are gradually introduced into a child's diet

1. Régulièrement, tout au long du suivi de mon enfant
2. Plusieurs fois pendant la période de diversification alimentaire
3. Uniquement au début de la diversification alimentaire
4. Jamais

1. Regularly, throughout the follow-up of my child
2. Several times during the period of complementary feeding
3. Only at the start of complementary feeding
4. Never

Q7. Depuis la naissance de votre plus jeune enfant, lors de rendez-vous médicaux habituels avec des professionnels de santé (pédiatre, médecin, infirmier…), leur avez-vous vous-même posé des questions ou demandé des conseils sur l'alimentation de votre enfant (aliments autre que le lait) dont la diversification alimentaire ?

La diversification alimentaire est la période pendant laquelle des aliments autres que le lait sont progressivement introduits dans l'alimentation d'un enfant

Q7. Since the birth of your youngest child, during usual medical appointments with health care professionals (pediatrician, doctor, nurse, etc.), did you yourself ask them questions or for advice on feeding your child (foods other than milk) including complementary feeding?

Complementary feeding is the period during which foods other than milk are gradually introduced into a child's diet

1. Régulièrement, tout au long du suivi de mon enfant
2. Plusieurs fois pendant la période de diversification alimentaire
3. Uniquement au début de la diversification alimentaire
4. Jamais

1. Regularly, throughout the follow-up of my child
2. Several times during the period of complementary feeding
3. Only at the start of complementary feeding
4. Never

Q8. Votre plus jeune enfant a-t-il ou a-t-il eu un problème de santé pouvant affecter fortement son alimentation, comme par exemple : reflux gastro œsophagien pris en charge médicalement, allergie aux protéines de lait de vache, intubation nasogastrique, anomalies congénitales du tube digestif ?

Q8. Does or did your youngest child have a health problem that could strongly affect his diet, such as: medically managed gastroesophageal reflux disease, cow's milk protein allergy, nasogastric intubation, congenital abnormalities of digestive the tract?

1. Oui
2. Non

1. Yes
2. No

Sentiment d'information vis-à-vis de l'alimentation des enfants de 0 à 3 ans
Perceptions on information about the feeding children 0 to 3 years old

Q9. Aujourd'hui, vous sentez-vous bien informé sur ce qu'un enfant de 0 à 3 ans peut manger en dehors du lait ?

Q9. Today, do you feel well informed about what a child aged between 0 and 3 years old can eat apart from milk?

1. Oui, tout à fait
2. Oui, plutôt
3. Non, plutôt pas
4. Non, pas du tout

1. Yes, absolutely
2. Yes, rather
3. No, rather not
4. No, not at all

Q10. Selon vous, l'alimentation de 0 à 3 ans (autre que le lait) est-elle importante pour la santé actuelle et future des enfants et leur croissance ?

Q10. In your opinion, is the topic of feeding children aged from 0 to 3 years old (foods other than milk) important for the present and future health of children and their growth?

1. Très importante
2. Plutôt importante
3. Plutôt pas importante
4. Pas du tout importante

1. Very important
2. Rather important
3. Rather not important
4. Not at all important

Q11. Selon vous, l'alimentation de 0 à 3 ans (autre que le lait) est-elle importante pour que les enfants aient ensuite de bonnes habitudes alimentaires ?

Q11. In your opinion, is the topic of feeding children aged from 0 to 3 years old (foods other than milk), important for children to develop healthy eating habits afterwards?

1. Très importante
2. Plutôt importante
3. Plutôt pas importante
4. Pas du tout importante

1. Very important
2. Rather important
3. Rather not important
4. Not at all important

Q12. Globalement, êtes-vous satisfait des informations sur l'alimentation de 0 à 3 ans (autre que le lait) dont la diversification alimentaire, que vous avez à votre disposition ?

La diversification alimentaire est la période pendant laquelle des aliments autres que le lait sont progressivement introduits dans l'alimentation d'un enfant

Q12. Overall, are you satisfied with the information on feeding children aged from 0 to 3 years old (foods other than milk), including complementary feeding, that you have at your disposal?

Complementary feeding is the period during which foods other than milk are gradually introduced into a child's diet

1. Très satisfait
2. Plutôt satisfait
3. Plutôt pas satisfait
4. Pas du tout satisfait

1. Very satisfied
2. Somewhat satisfied
3. Rather not satisfied
4. Not at all satisfied

Q13. Et globalement concernant l'alimentation de 0 à 3 ans (autre que le lait) dont la diversification alimentaire, diriez-vous que les informations et conseils que vous avez à votre disposition sur ce sujet…

La diversification alimentaire est la période pendant laquelle des aliments autres que le lait sont progressivement introduits dans l'alimentation d'un enfant

Q13. And generally concerning the topic of feeding children aged from 0 to 3 years old (foods other than milk), would you say that the information and advice you have at your disposal on this subject ...

Complementary feeding is the period during which foods other than milk are gradually introduced into a child's diet

a) Répondent à vos questions
b) Sont clairs, faciles à comprendre

c) Sont faciles à mettre en pratique pour votre enfant
d) Se contredisent, ne sont pas cohérents entre eux
e) Sont culpabilisants pour les parents
   1. Oui, tout à fait
   2. Oui, plutôt
   3. Non, plutôt pas
   4. Non, pas du tout

a) Answer to your questions
b) Are clear, easy to understand
c) Are easy to put into practice for your child
d) Contradict each other, are not consistent with each other
e) Are guilty-laden for parents
   1. Yes, absolutely
   2. Yes, rather
   3. No, rather not
   4. No, not at all

Q14. Êtes-vous d'accord ou non avec les affirmations suivantes concernant la diversification alimentaire ?

La diversification alimentaire est la période pendant laquelle des aliments autres que le lait sont progressivement introduits dans l'alimentation d'un enfant

Q14. Do you agree or disagree with the following statements about complementary feeding?

Complementary feeding is the period during which foods other than milk are gradually introduced into a child's diet

a) Il est facile de trouver des informations concernant la diversification alimentaire
b) La diversification alimentaire de mon plus jeune enfant est ou a été source d'inquiétude
c) La diversification alimentaire pour mon plus jeune enfant se passe bien ou s'est bien passée
d) La diversification alimentaire est plus facile pour mon dernier enfant que pour le premier
   1. Tout à fait d'accord
   2. Plutôt d'accord
   3. Plutôt pas d'accord
   4. Pas du tout d'accord

a) It is easy to find information about complementary feeding
b) Complementary feeding for my youngest child is or was a source of concern (only for multiparous)
c) Complementary feeding for my youngest child is going well or has gone well
d) Complementary feeding is easier for my last child than for my first child
   1. Strongly agree
   2. Tend to agree
   3. Tend to disagree
   4. Strongly disagree

Sources d'informations et recherche
Sources of information

Q15. Au cours des 12 derniers mois, par quels moyens avez-vous eu des informations sur l'alimentation de votre plus jeune enfant (alimentation autre que le lait) ?

Plusieurs réponses possibles

Q15. During the past 12 months, how did you learn about the topic of feeding children aged from 0 to 3 years old (foods other than milk)?

Several answers are possible

Média

1. Télévision
2. Radio

Support ou document papier

3. Presse écrite, journaux, magazines
4. Livres

Internet, réseaux sociaux ou applications smartphone

5. Blog ou forum de parents
6. Application pour smartphone
7. Site internet d'industriel ou fabricant d'aliments pour bébé (bledina, nestlé, babybio, hipp, goodgout…)
8. Site internet spécialisé dans la petite enfance (parents.fr, magicmaman.com…)
9. Site internet dédié à la santé (doctissimo.fr, santémagazine.fr, topsanté.com, passeportsanté.net…)
10. Site internet d'une autorité de santé (ameli.fr, mangerbouger.fr, santépubliquefrance.fr, santé.gouv.fr, santé.fr…)
11. Site internet d'une mutuelle ou d'une assurance (lamutuellegenerale.fr, mutualite.fr, axaprevention.fr, harmonie-mutuelle.fr, interiale.fr…)
12. Réseaux sociaux (facebook, instagram, twitter, youtube, whatsapp…)
13. Autre site internet

Entourage

14. Mes amis
15. Mes parents
16. Mes grands-parents
17. Un autre membre de la famille

Professionnel de santé

18. Médecin généraliste
19. Pédiatre
20. Infirmier(ère), puériculteur(trice)
21. Diététicien(ne)

22. Sage-femme

Professionnel de la petite enfance

23. Personnel de PMI ou d'établissement d'accueil de jeunes enfants (crèche, accueil collectif…)
24. Assistante maternelle

25. Autre moyen, précisez : ____________________

Media

1. Television
2. Radio

Paper support or document

3. Written press, newspapers, magazines
4. Books

Internet, social networks or smartphone applications

5. Parents' blog or forum
6. Smartphone application
7. Industrial or baby food manufacturer's website (bledina, nestlé, babybio, hipp, goodgout, etc.)
8. Website specializing in early childhood (parents.fr, magicmaman.com…)
9. Internet site dedicated to health (doctissimo.fr, santémagazine.fr, topsanté.com, passportsanté.net, etc.)
10. Website of a health authority (ameli.fr, mangerbouger.fr, santépubliquefrance.fr, santé.gouv.fr, santé.fr, etc.)
11. Website of a mutual or insurance company (lamutuellegenerale.fr, mutualite.fr, axaprevention.fr, harmonie-mutuelle.fr, interiale.fr ...)
12. Social networks (facebook, instagram, twitter, youtube, whatsapp…)
13. Other website

Parental network

14. My friends
15. My parents
16. My grandparents
17. Another family member

Health professional

18. General practitioner
19. Pediatrician
20. Nurse, childcare worker
21. Dietitian
22. Midwife

Early childhood professional

23. Staff of PMI or establishment for the reception of young children (crèche, collective reception, etc.)

24. Maternal assistant

25. Other means, specify: ____________________

Q16. Sur quel(s) réseau(x) social(ux) vous êtes-vous informé concernant l'alimentation de votre plus jeune enfant (aliments autres que le lait) ?

Plusieurs réponses possibles

Q16. On which social network(s) did you learn about the topic of feeding children aged from 0 to 3 years old (foods other than milk) for your youngest child?

It is possible to give more than one answer

1. Facebook
2. Instagram
3. Twitter
4. Youtube
5. Whatsapp
6. Autre

Q17. Vous arrive-t-il de partager sur les réseaux sociaux, sur des blogs ou des forums, des informations sur l'alimentation de l'enfant de 0 à 3 ans que vous trouviez utiles ?

Q17. Do you ever share on social media, blogs or forums information that you find useful about feeding children aged from 0 to 3 years old (foods other than milk)?

1. Très souvent
2. Souvent
3. De temps en temps
4. Jamais

1. Very often
2. Often
3. From time to time
4. Never

Q18. Pour chacune de vos sources d'information, indiquez sur une échelle de 1 à 10 dans quelle mesure elles ont influencé vos décisions sur la façon de nourrir votre plus jeune enfant au cours des 12 derniers mois.

1 signifie qu'elle n'a pas du tout influencé vos décisions et 10 signifie qu'elle a très fortement influencé vos décisions. Les notes intermédiaires permettent de nuancer votre jugement.

Q18. For each of the source of information you have used, rate on a scale of 1 to 10 how much it influenced your decisions about how to feed your youngest child during the past 12 months.

1 means it did not influence your decisions at all and 10 means it very strongly influenced your decisions. The intermediate points of the scale allow you to qualify your judgment.

a) La télévision ou la radio
b) Les supports ou documents papier comme la presse écrite, les journaux, les magazines ou les livres
c) Internet, réseaux sociaux ou applications smartphone
d) L'entourage
e) Les professionnels de santé
f) Les professionnels de la petite enfance

a) Television or radio
b) Media or paper documents such as the written press, newspapers, magazines or books
c) Internet, social networks or smartphone applications
d) Parental network
e) Health care professionals
f) Early childhood professionals

N'a pas du tout influencé mes décisions											A très fortement influencé mes décisions
	1	2	3	4	5	6	7	8	9	10	

It did not influence my decisions at all											It has influenced my decisions very strongly
	1	2	3	4	5	6	7	8	9	10	

Q19. Vous est-il arrivé de chercher des informations concernant l'alimentation des enfants de 0 à 3 ans (autre que le lait) pour votre enfant ?
Q19. Have you ever looked for information about the topic of feeding children aged from 0 to 3 years old (foods other than milk) for your child?

1. Oui
2. Non

1. Yes
2. No

Q20. Quel âge avait votre plus jeune enfant lorsque vous avez commencé à chercher des informations sur l'alimentation autre que le lait ?
S'il avait moins d'1 mois, indiquer 0

Q20. How old was your youngest child when you first started looking for information about food other than milk?

If he was less than 1 month old, enter 0

/__/__/ mois

/__/__/ months

**Confiance dans les sources d'informations**<br>**Trust in sources of information**

Q21. Pour avoir une information fiable sur l'alimentation des enfants de 0 à 3 ans (autre que le lait), faites-vous personnellement confiance ou non…

Q21. To have reliable information on the topic of feeding children aged from 0 to 3 years old (foods other than milk), do you personally trust or not ...

a) Aux médias comme la télévision, la radio ou la presse écrite
b) Aux livres dédiés à la petite enfance
c) À Internet
d) Aux réseaux sociaux, blogs ou forums de parents
e) Aux autorités de santé ou pouvoirs publics
f) Aux industriels ou fabricants de produits alimentaires pour jeunes enfants
g) Aux professionnels de santé (médecin, pédiatre, infirmière, sage-femme…)
h) Aux professionnels de la petite enfance (personnel de PMI ou d'établissement d'accueil des jeunes enfants, assistante maternelle…)

1. Tout à fait confiance
2. Plutôt confiance
3. Plutôt pas confiance
4. Pas du tout confiance

a) Media such as television, radio or the written press
b) Books dedicated to early childhood / parenting
c) Internet
d) Social networks, blogs or parent forums
e) Public health authorities
f) Manufacturers of food products for young children
g) Health care professionals (doctor, pediatrician, nurse, midwife, etc.)
h) Early childhood professionals (staff from PMI or childcare facilities, childminders, etc.)

1. Complete trust
2. Somewhat trust
3. Tend not to trust
4. Not trust at all

**Attentes en matière de type et de format d'informations**

Expectations for the type and format of information

Q22. Quels types d'informations sur l'alimentation des enfants de 0 à 3 ans (autre que le lait) et la diversification alimentaire vous seraient particulièrement utiles pour votre enfant le plus jeune ?

5 réponses maximum

Q22. Which kinds of information about the topic of feeding children aged from 0 to 3 years old (foods other than milk) and complementary feeding would be particularly useful for your youngest child?

5 answers maximum

1. Âge d'introduction des premiers aliments autres que le lait
2. Âge d'introduction des différents groupes d'aliments
3. Âge et modalités d'introduction des premiers morceaux d'aliments
4. Comment donner ou présenter les aliments en cas de refus
5. Quantités et tailles des portions à proposer à l'enfant
6. Stratégies alimentaires en cas de « petit appétit » de l'enfant
7. Stratégies alimentaires en cas de « gros appétit » de l'enfant
8. Comment interpréter les signaux de l'enfant lorsqu'il mange ou refuse de manger
9. Comment nourrir un enfant pour favoriser le développement d'habitudes alimentaires saines
10. Exemples de recettes
11. Exemples de menus
12. Adaptation de la quantité de lait à l'introduction des autres aliments
13. Autres informations, précisez :__________

1. Age of introduction of the first foods other than milk
2. Age of introduction of the different food groups
3. Age and method of introduction of the first pieces of food
4. How to give or present the food in case of refusal
5. Quantities and sizes of portions to offer to the child
6. Feeding strategies in the event of a child's "small appetite"
7. Feeding strategies in the event of a child's "big appetite"
8. How to interpret the child's hunger and satiety cues
9. How to feed a child to support the development of healthy eating habits
10. Examples of recipes
11. Examples of menus
12. Adaptation of the quantity of milk to the introduction of other foods
13. Other information, specify: __________

Q23. Pour vous, quels types de supports sont les plus adaptés pour avoir des informations sur l'alimentation des enfants de 0 à 3 ans (autre que le lait) dont la diversification alimentaire ?

3 réponses maximum

Q23. For you, which types of media are the most suitable for obtaining information on the topic of feeding children aged from 0 to 3 years old (foods other than milk), including complementary feeding?

3 answers maximum

1. Une brochure papier d'1 page
2. Un livret papier de plusieurs pages
3. Un site internet
4. Une application pour smartphone
5. Un espace interactif sur internet avec la possibilité de poser des questions (chat, blog, forum, post de témoignage…)
6. Autre, précisez :__________

1. A 1-page paper brochure
2. A paper booklet of several pages
3. A website
4. A smartphone application
5. An interactive space on the internet with the possibility of asking questions (chat, blog, forum, testimonial post, etc.)

6. Other, specify: __________

Autres renseignements signalétiques<br>Other socio demographic information

Q24. Êtes-vous né en France ?

Q24. Were you born in France?

1. Oui
2. Non

1. Yes
2. No

Q25. Depuis combien de temps habitez-vous en France ?

Si vous habitez en France depuis moins d'un an, indiquez 0

Q25. How long have you lived in France?

If you have lived in France for less than a year, enter 0

/__/__/ ans

/__/__/ years

Q26. Le français est-il votre langue maternelle ?

Q26. Is French your mother tongue?

1. Oui
2. Non

1. Yes
2. No

Q27. Quel est le diplôme le plus élevé que vous ayez obtenu ?

Q27. What is the highest degree that you have obtained?

1. Aucun diplôme
2. Certificat d'études primaires ou diplôme étranger de même niveau
3. CAP, BEP ou diplôme de ce niveau
4. Brevet des collèges, BEPC, brevet élémentaire ou diplôme étranger de même niveau
5. Baccalauréat technologique ou professionnel ou diplôme de ce niveau dont BT et BTA…
6. Baccalauréat général, brevet supérieur, capacité en droit, DAEU, diplôme étranger de ce niveau
7. Baccalauréat sans autre précision
8. Diplôme de niveau bac+2 (DEUG, DUT, BTS)
9. Diplôme de niveau bac+3 ou bac+4 (2nd cycle, master 1, licence ou maîtrise)
10. Diplôme de niveau bac+5 ou supérieur (3ème cycle, master 2, DESS, DEA, diplôme d'ingénieur, de grande école, doctorat…)

1. I do not have any diploma
2. Primary school certificate or foreign diploma of the same level
3. CAP, BEP or diploma of this level
4. College diploma, BEPC, elementary certificate or foreign diploma of the same level
5. Technological or professional baccalaureate or diploma of this level including BT and BTA…
6. General baccalaureate, higher certificate, capacity in law, DAEU, foreign diploma of this level
7. Baccalaureate without further clarification
8. Bac + 2 level diploma (DEUG, DUT, BTS)
9. Bac + 3 or bac + 4 level diploma (2nd cycle, master 1, license or master's degree)
10. Bac + 5 or higher level diploma (3rd cycle, master's 2, DESS, DEA, engineering diploma, high school, doctorate ...)

Q28. Actuellement, diriez-vous qu'au sein de votre foyer, financièrement…

Q28. Currently, would you say that within your household, financially ...

1. Vous êtes à l'aise
2. Ça va
3. C'est juste, il faut faire attention

4. Vous y arrivez difficilement
5. Vous ne pouvez pas y arriver sans faire de dettes
6. Je ne souhaite pas répondre

1. You are comfortable
2. It's okay
3. It's fair, you have to be careful
4. You hardly get there
5. You can't do it without getting into debt
6. I do not wish to answer
