# Supplementary material for: The Perceptions and Needs of French Parents and Pediatricians Concerning Information on Complementary Feeding
Source: Nutrients. 2021 Jun 22;13(7):2142. doi: 10.3390/nu13072142 (PMC8308433; doi:10.3390/nu13072142)
Supplement: Supplementary file 1 [file nutrients-13-02142-s001.zip › Supplement Material 2_ped survey.pdf]

Supplemental Material 2. Pediatricians' original questionnaire with English translation in blue.

Professionnels de santé
« La diversification alimentaire »
Healthcare professionals
"Complementary feeding"

Dans le cadre d'un programme de recherche mené par l'INRA, ce questionnaire a pour objectif de décrire la manière dont les professionnels de santé abordent la diversification alimentaire avec les parents. Ce sondage ne devrait vous prendre que 15 minutes. Vos réponses seront anonymes et votre participation est entièrement libre. D'avance, nous vous remercions de votre aide.

Pour commencer, cliquez s'il vous plait sur le bouton "Suivant".

Veuillez cocher pour indiquer votre réponse.

As part of a research program led by INRA, this questionnaire aims to describe the way in which healthcare professionals approach complementary feeding with parents. This survey should only take you 15 minutes to complete. Your answers will be anonymous and your participation is completely free. In advance, we thank you for your help.

To get started, please click on the "Next" button.

Please tick to indicate your answer.

1. Données démographiques
1. Demographic data

1.1	Quelle est votre profession ?<br><br>What is your profession ?	☐ Médecin généraliste<br>☐ Pédiatre<br>☐ Infirmier/ère puériculteur/trice<br>☐ Personnel de PMI ou d'établissement d'accueil des jeunes enfants : auxiliaire de puériculture, aide soignant, éducateur de jeunes enfants, autre<br>☐ Diététicien/ne<br>☐ Sage-femme/Maïeuticien<br>☐ Autre (veuillez préciser)<br>……………………………………

		☐ General practitioner<br>☐ Pediatrician<br>☐ Pediatric nurse<br>☐ PMI or young childcare establishment staff: childcare assistant, caregiver, educator of young children, other<br>☐ Dietitian<br>☐ Midwife / Maieutician<br>☐ Other (please specify)<br>…………………………………
1.2	Depuis combien d'années exercez-vous cette profession ?<br><br>Since how many years have you practiced this profession?	☐ 0-10 ans<br>☐ 11-20 ans<br>☐ 21-30 ans<br>☐ >30 ans<br>☐ 0-10 years<br>☐ 11-20 years<br>☐ 21-30 years<br>☐ >30 years
1.3	Quel âge avez-vous ?<br><br>How old are you?	☐ ≤ 30 ans<br>☐ 31-40 ans<br>☐ 41-50 ans<br>☐ 51-60 ans<br>☐ ≥ 61 ans<br>☐ ≤ 30 years old<br>☐ 31-40 years old<br>☐ 41-50 years old<br>☐ 51-60 years old<br>☐ ≥ 61 years old
1.4	Êtes-vous... ?<br><br>Are you…?	☐ un homme<br>☐ une femme<br>☐ man<br>☐ woman
1.5	Où travaillez-vous ?<br><br>Where do you work?	☐ zone urbaine<br>☐ zone rurale<br>☐ Urban area<br>☐ Rural area
1.6	Quel est votre code postal ?<br><br>What is your postal code?	……..

1.7	Avez-vous vous même des enfants ?<br><br>Do you have children?	☐ Oui ☐ Non<br>☐ Yes ☐ No
1.8	En quelles années sont nés vos premiers et derniers enfants ?<br><br>In which years were your first and last children born?	1er enfant ____<br>Dernier enfant ____<br>First child ____<br>Last child ____

2. Attitudes et perceptions

2. Attitudes and perceptions

	Indiquez dans quelle mesure vous êtes d'accord avec les affirmations suivantes<br>*Indicate the extent to which you agree with the following statements*	
2.1	Conseiller les parents sur la diversification alimentaire est l'une de mes responsabilités.<br><br>Advising parents on complementary feeding is one of my responsibilities.	☐ Tout à fait d'accord<br>☐ D'accord<br>☐ Ni en désaccord, ni d'accord<br>☐ Pas d'accord<br>☐ Pas du tout d'accord<br>☐ Totally agree<br>☐ Agree<br>☐ Neither disagree nor agree<br>☐ Disagree<br>☐ Strongly disagree
2.2	La diversification alimentaire est une étape importante pour le développement équilibré des nourrissons.<br><br>Complementary feeding is an important step for the balanced development of infants.	☐ Tout à fait d'accord<br>☐ D'accord<br>☐ Ni en désaccord, ni d'accord<br>☐ Pas d'accord<br>☐ Pas du tout d'accord<br>☐ Totally agree<br>☐ Agree<br>☐ Neither disagree nor agree<br>☐ Disagree<br>☐ Strongly disagree
2.3	La diversification alimentaire est une étape importante pour l'acquisition de bonnes habitudes alimentaires chez les nourrissons.<br><br>Complementary feeding is an important step in the acquisition of healthy eating habits in infants.	☐ Tout à fait d'accord<br>☐ D'accord<br>☐ Ni en désaccord, ni d'accord<br>☐ Pas d'accord<br>☐ Pas du tout d'accord<br>☐ Totally agree<br>☐ Agree<br>☐ Neither disagree nor agree

		☐ Disagree<br>☐ Strongly disagree
2.4	Conseiller les parents sur la diversification alimentaire est efficace pour leur faire adopter des pratiques alimentaires saines pour leur enfant.<br><br>Counseling parents on complementary feeding is effective in making them adopt healthy eating practices for their child.	☐ Tout à fait d'accord<br>☐ D'accord<br>☐ Ni en désaccord, ni d'accord<br>☐ Pas d'accord<br>☐ Pas du tout d'accord<br>☐ Totally agree<br>☐ Agree<br>☐ Neither disagree nor agree<br>☐ Disagree<br>☐ Strongly disagree
2.5	Je me sens à l'aise pour conseiller les parents sur la diversification alimentaire.<br><br>I feel comfortable advising parents on complementary feeding.	☐ Tout à fait d'accord<br>☐ D'accord<br>☐ Ni en désaccord, ni d'accord<br>☐ Pas d'accord<br>☐ Pas du tout d'accord<br>☐ Totally agree<br>☐ Agree<br>☐ Neither disagree nor agree<br>☐ Disagree<br>☐ Strongly disagree
2.6	Je pense que les parents veulent plus d'informations sur la diversification alimentaire que ce que je suis en mesure de leur fournir.<br><br>I think parents want more information on complementary feeding than what I can provide them with.	☐ Tout à fait d'accord<br>☐ D'accord<br>☐ Ni en désaccord, ni d'accord<br>☐ Pas d'accord<br>☐ Pas du tout d'accord<br>☐ Totally agree<br>☐ Agree<br>☐ Neither disagree nor agree<br>☐ Disagree<br>☐ Strongly disagree
2.7	Je pense avoir suffisamment de connaissances sur la nutrition infantile et la diversification alimentaire.<br><br>I think I have enough knowledge about infant nutrition and complementary feeding.	☐ Tout à fait d'accord<br>☐ D'accord<br>☐ Ni en désaccord, ni d'accord<br>☐ Pas d'accord<br>☐ Pas du tout d'accord<br>☐ Totally agree<br>☐ Agree<br>☐ Neither disagree nor agree

		☐ Disagree<br>☐ Strongly disagree
2.8	Au cours des dernières années, avez-vous acquis de nouvelles connaissances sur la diversification alimentaire ?<br><br>Over the past years, did you acquire new knowledge regarding complementary feeding?	☐ Oui<br>☐ Non<br>☐ Yes<br>☐ No
2.9	Au cours des deux dernières années, comment avez-vous acquis de nouvelles connaissances sur la diversification alimentaire ?<br>(plusieurs réponses possibles)<br><br>Over the past two years, how did you acquire new knowledge about complementary feeding?<br>(multiple answers allowed)	☐ Formation professionnelle continue: réunions, congrès et/ou medias (journaux professionnels, sites internet professionnels, newsletters, etc.)<br>☐ Brochures de santé publique (Santé Publique France, ARS, IREPS…)<br>☐ Brochures des fabricants de laits ou aliments pour bébés<br>☐ Événements organisés par des fabricants de laits ou aliments pour bébés<br>☐ Blogs sur la nutrition, autres sites des réseaux de nutrition<br>☐ Médias généralistes (radio, télévision, journaux, magazines)<br>☐ Discussions avec des collègues<br>☐ Autre (veuillez préciser)<br>……………………………………..<br>☐ Continuing professional training : meetings, congresses and / or media (professional journals, professional websites, newsletters, etc.)<br>☐ Public health brochures (Public Health France, ARS, IREPS, etc.)<br>☐ Brochures from manufacturers of baby milk or food<br>☐ Events organized by manufacturers of milk or baby food

		☐ Nutrition blogs, other nutrition network sites<br>☐ General media (radio, television, newspapers, magazines)<br>☐ Discussions with colleagues<br>☐ Other (please specify)<br>…………………………………… ..
2.10	Parmi les propositions suivantes, quelles sont selon vous les organismes chargés de formuler les recommandations officielles concernant la diversification alimentaire ?<br><br>Among the following propositions, which organizations do you think are responsible for formulating official recommendations concerning complementary feeding?	☐ Santé publique France<br>☐ Sociétés savantes (Société française de Pédiatrie, Association de Pédiatrie Ambulatoire, Société Française de Nutrition, Académie de Médecine, autre)<br>☐ ARS (Agence régionale de santé) / IREPS (Instance régionale d'Education et de Promotion de la Santé)<br>☐ ANSES (Agence nationale de sécurité sanitaire de l'alimentation, de l'environnement et du travail)<br>☐ Fabricants de laits et d'aliments pour bébés<br>☐ Médias généralistes (radio, télévision, journaux, magazines)<br>☐ Instituts de recherche / Universités<br>☐ Public health France<br>☐ Learned societies (French Society of Pediatrics, Association of Ambulatory Pediatrics, French Society of Nutrition, Academy of Medicine, other)<br>☐ ARS (Regional Health Agency) / IREPS (Regional Authority for Education and Health Promotion)<br>☐ ANSES (National Agency for Food, Environmental and Occupational Health Safety)<br>☐ Milk and baby food manufacturers

		☐ General media (radio, television, newspapers, magazines)<br>☐ Research institutes / Universities
2.11	Hiérarchiser les freins pour donner des conseils aux parents sur la diversification alimentaire listés ci-dessous du plus contraignant au moins contraignant<br>- le manque de temps<br>- mes compétences en conseil<br>- le manque d'intérêt de certains parents<br>- le manque de matériel de communication approprié<br><br>Prioritize the obstacles to give advice to parents on complementary feeding listed below from the most restrictive to the least restrictive<br>- lack of time<br>- my consulting skills<br>- the lack of interest of some parents<br>- lack of appropriate communication material	

3. Vos pratiques actuelles en matière de communication avec les parents

3. Your current communication practices with parents

3.1	Sur l'ensemble de vos consultations/RDV avec des parents de jeunes enfants (0-3 ans), à quelle fréquence conseillez-vous les parents sur la diversification alimentaire ?<br><br>Over all of your consultations / appointments with parents of young children (0-3 years old), how often do you advise parents on complementary feeding?	☐ Jamais<br>☐ Uniquement si j'identifie que cela est nécessaire<br>☐ Toujours<br>☐ Never<br>☐ Only if I identify that it is necessary<br>☐ Always
3.2	Quel âge ont habituellement les nourrissons lorsque vous commencez à parler de la diversification alimentaire ?<br><br>How old are infants usually when you start talking about complementary feeding?	☐ Avant la naissance<br>☐ Peu après la naissance<br>☐ 1 mois ☐ 2 mois<br>☐ 3 mois ☐ 4 mois<br>☐ 5 mois ☐ 6 mois<br>☐ 9 mois ☐ 12 mois<br>☐ Before birth<br>☐ Soon after birth<br>☐ 1 month ☐ 2 months<br>☐ 3 months ☐ 4 months<br>☐ 5 months ☐ 6 months<br>☐ 9 months ☐ 12 months

3.3	Quel âge ont habituellement les nourrissons lorsque vous parlez des étapes de la diversification alimentaire (introduction des groupes d'aliments,…) ?<br><br>How old are infants usually when you talk about the stages of complementary feeding (introduction of food groups, etc.)?	☐ 1 mois ☐ 2 mois<br>☐ 3 mois ☐ 4 mois<br>☐ 5 mois ☐ 6 mois<br>☐ 9 mois ☐ 12 mois<br>☐ 16 mois ☐ 20 mois<br>☐ 24 mois ☐ 30 mois<br>☐ 36 mois<br>☐ 1 month ☐ 2 months<br>☐ 3 months ☐ 4 months<br>☐ 5 months ☐ 6 months<br>☐ 9 months ☐ 12 months<br>☐ 16 months ☐ 20 months<br>☐ 24 months ☐ 30 months<br>☐ 36 months
3.4	Avec qui discutez-vous de la diversification alimentaire le plus souvent ?<br>(plusieurs réponses possibles)<br><br>Who do you the most often discuss complementary feeding with?<br>(multiple answers allowed)	☐ Mère ☐ Père<br>☐ Autres membres de la famille (veuillez préciser)<br>……………………………………..<br>☐ Mother ☐ Father<br>☐ Other family members (please specify)<br>……………………………………… ..
3.5	De quelle manière abordez-vous la diversification alimentaire ?<br>(plusieurs réponses possibles)<br><br>How do you approach the topic of complementary feeding?<br>(multiple answers allowed)	☐ En discutant<br>☐ En remettant des documents<br>☐ En conseillant des sites internet<br>☐ En m'appuyant sur le carnet de santé<br>☐ Autre (veuillez préciser)<br>☐ By discussing<br>☐ By handing over documents<br>☐ By advising websites<br>☐ By relying on the health notebook<br>☐ Other (please specify)
3.6	À quelle fréquence donnez-vous des documents concernant la diversification alimentaire aux parents ?<br><br>How often do you give documents about complementary feeding to parents?	☐ Jamais<br>☐ Rarement<br>☐ Parfois<br>☐ Souvent<br>☐ Toujours<br>☐ Never<br>☐ Rarely

		☐ Sometimes<br>☐ Often<br>☐ Always
3.7	Qui vous fournit les documents de communication sur la diversification alimentaire à remettre aux parents ?<br>(plusieurs réponses possibles)<br><br>Who provides you with the communication documents on complementary feeding to give to parents?<br>(more than one answer allowed)	☐ Santé publique France<br>☐ PNNS<br>☐ ARS<br>☐ IREPS<br>☐ CAF<br>☐ Sociétés de pédiatrie (Société Française de Pédiatrie, Association de Pédiatrie Ambulatoire, Société Française de Nutrition, Académie de Médecine, autre)<br>☐ Fabricants de laits et d'aliments pour bébés<br>☐ Fabricants de matériel de puériculture (ex : tire-lait)<br>☐ Conseil général<br>☐ Associations<br>☐ Je crée mes propres documents<br>☐ Autre (veuillez préciser)<br>……………………………………..<br>☐ Public health France<br>☐ PNNS<br>☐ ARS<br>☐ IREPS<br>☐ CAF<br>☐ Pediatric societies (French Society of Pediatrics, Association of Outpatient Pediatrics, French Society of Nutrition, Academy of Medicine, other)<br>☐ Milk and baby food manufacturers<br>☐ Manufacturers of childcare equipment (e.g. breast pump)<br>☐ General Council<br>☐ Associations<br>☐ I create my own documents<br>☐ Other (please specify)<br>…………………………………… ..
	Indiquez dans quelle mesure vous êtes d'accord avec les affirmations suivantes	

	Indicate the extent to which you agree with the following statements	
3.8	Je suis satisfait des documents sur la diversification alimentaire dont je dispose.<br><br>I am satisfied with the documents on complementary feeding that I have.	☐ Tout à fait d'accord<br>☐ D'accord<br>☐ Ni en désaccord ni d'accord<br>☐ Pas d'accord<br>☐ Pas du tout d'accord<br>☐ Totally agree<br>☐ Agree<br>☐ Neither disagree nor agree<br>☐ Disagree<br>☐ Strongly disagree
3.9	Je pense que les documents que je remets aux parents suscitent leur intérêt pour la diversification alimentaire.<br><br>I think that the documents that I give to parents arouse their interest in complementary feeding.	☐ Tout à fait d'accord<br>☐ D'accord<br>☐ Ni en désaccord, ni d'accord<br>☐ Pas d'accord<br>☐ Pas du tout d'accord<br>☐ Totally agree<br>☐ Agree<br>☐ Neither disagree nor agree<br>☐ Disagree<br>☐ Strongly disagree
3.10	Je pense que les documents que je remets aux parents permettent de tenir compte des différences culturelles et/ou financières propres à chaque famille.<br><br>I think that the documents that I give to parents make it possible to take into account the cultural and / or financial differences specific to each family.	☐ Tout à fait d'accord<br>☐ D'accord<br>☐ Ni en désaccord, ni d'accord<br>☐ Pas d'accord<br>☐ Pas du tout d'accord<br>☐ Totally agree<br>☐ Agree<br>☐ Neither disagree nor agree<br>☐ Disagree<br>☐ Strongly disagree

4. Nouveaux plans et suggestions
4. New plans and suggestions

4.1	Hiérarchiser les caractéristiques suivantes de la plus importante à la moins importante pour des documents à destination des parents afin d’attirer leur attention sur la diversification alimentaire.	Le matériel que je donne devrait être :<br>☐ Clair<br>☐ Synthétique<br>☐ Détaillé<br>☐ Axé sur les aspects pratiques

<table>
<tr><td></td><td>Prioritize the following characteristics from the most important to the least important for documents intended for parents in order to draw their attention to complementary feeding.</td><td>☐ Facile à consulter
☐ Illustré
The material I donate should be:
☐ Clear
☐ Synthetic
☐ Detailed
☐ Focused on practical aspects
☐ Easy to consult
☐ Illustrated</td></tr>
<tr><td>4.2</td><td colspan="2">Concernant les informations à transmettre aux parents à propos de la diversification alimentaire, merci de cocher si les sujets sont très importants ou moins importants selon vous.

Regarding the information to be transmitted to parents about complementary feeding, please check if the subjects are very important or less important in your opinion.
<table>
<tr><th>Sujets concernant la diversification alimentaire
Topics concerning complementary feeding</th><th>Sujets très importants
Very important topic</th><th>Sujets moins importants
Less important topics</th></tr>
<tr><td>Age de début de la diversification alimentaire
Age of start of complementary feeding</td><td></td><td></td></tr>
<tr><td>Age d’introduction des différents groupes d’aliments
Age and modalities of introduction of different food groups</td><td></td><td></td></tr>
<tr><td>Age d’introduction des aliments texturés et progression souhaitée
Age and modalities of introduction of first food pieces and different textures</td><td></td><td></td></tr>
<tr><td>Stratégies de présentation en cas de refus alimentaire
How to present food in case of refusal</td><td></td><td></td></tr>
<tr><td>Quantités et tailles des portions
Portions sizes of food and milk</td><td></td><td></td></tr>
<tr><td>Stratégies alimentaires en cas de « petit mangeur »
How to deal with “little” appetite</td><td></td><td></td></tr>
<tr><td>Stratégies alimentaires en cas de « gros mangeur »
How to deal with “big” appetite</td><td></td><td></td></tr>
<tr><td>Observations du comportement des nourrissons
How to interpret the child’s behavior</td><td></td><td></td></tr>
<tr><td>Comment nourrir un enfant pour favoriser le développement de saines habitudes alimentaires</td><td></td><td></td></tr>
</table></td></tr>
</table>

How to feed a child to promote the development of healthy eating habits		
Exemples de menus ou de recettes<br>Examples of menus and recipes		

4.3 Concernant les informations à transmettre aux parents à propos de la diversification alimentaire, merci de cocher si vous voulez plus d’information ou si vous avez déjà suffisamment d'information en ce qui concerne les sujets suivants.

Regarding the information to be transmitted to parents about complementary feeding, please check if you want more information or if you already have enough information regarding the following topics.

**Sujets concernant la diversification alimentaire**<br>**Topics concerning complementary feeding**	**Sujets pour lesquels j'ai besoin de plus d'information**<br>**Topics for which I need more information**	**Sujets pour lesquels j'ai déjà suffisamment d'information**<br>**Topics for which I already have enough information**
Age de début de la diversification alimentaire<br>Age of start of complementary feeding		
Age d’introduction des différents groupes d’aliments<br>Age and modalities of introduction of different food groups		
Age d’introduction des aliments texturés et progression souhaitée<br>Age and modalities of introduction of first food pieces and different textures		
Stratégies de présentation en cas de refus alimentaire<br>How to present food in case of refusal		
Quantités et tailles des portions<br>Portions sizes of food and milk		
Stratégies alimentaires en cas de « petit mangeur »<br>How to deal with “little” appetite		
Stratégies alimentaires en cas de « gros mangeur »<br>How to deal with “big” appetite		

	Observations du comportement des nourrissons<br>How to interpret the child's behavior		
	Comment nourrir un enfant pour favoriser le développement de saines habitudes alimentaires<br>How to feed a child to promote the development of healthy eating habits		
	Exemples de menus ou de recettes<br>Examples of menus and recipes		
4.4	En dehors des discussions que vous pouvez mener en face à face, quels sont les outils de communication que vous considérez comme étant les plus accessibles et plus susceptibles d'attirer l'attention des parents aujourd'hui ?<br>(plusieurs réponses possibles)<br><br>Aside from face-to-face discussions, which communication tools do you consider the most accessible and most likely to grab the attention of parents today?<br>(more than one answer allowed)	☐ Brochures<br>☐ Télévision<br>☐ Radio<br>☐ Sites internet<br>☐ YouTube<br>☐ Réseaux sociaux (Facebook, Instagram, Twitter, Pinterest)<br>☐ Applications pour smartphone<br>☐ Blogs<br>☐ Forums de discussion<br>☐ Chats (Des chats individuels offrent la possibilité d'échanger en temps réel et en privé avec un intervenant)<br>☐ Coups de téléphone à des amis ou parents / grands-parents<br>☐ Brochures<br>☐ Television<br>☐ Radio<br>☐ Websites<br>☐ YouTube<br>☐ Social networks (Facebook, Instagram, Twitter, Pinterest)<br>☐ Smartphone applications<br>☐ Blogs<br>☐ Discussion forums<br>☐ Chats (Individual chats offer the possibility of discussing in real time and in private with a speaker)<br>☐ Phone calls to friends or relatives / grandparents	
4.5	Avez-vous d'autres suggestions (en termes de contenu et de format) concernant les outils de	……………………………………..<br>..………………………………............	

	communication sur la diversification alimentaire à fournir aux parents ?<br><br>Do you have any other suggestion (in terms of content and format) concerning the communication tools on complementary feeding to be provided to parents?	..…………………………………..

Question facultative : Avez-vous d'autres suggestions (en termes de contenu et de format) concernant les outils de communication sur la diversification alimentaire à fournir aux parents ?

Optional question: Do you have any other suggestion (in terms of content and format) concerning the communication tools on complementary feeding to be provided to parents?

………

………

Merci d'avoir pris le temps de remplir ce questionnaire !

Thank you for taking the time to complete this questionnaire!
